# Factors influencing the length of stay in forensic psychiatric settings: a systematic review

**DOI:** 10.1186/s12913-024-10863-x

**Published:** 2024-03-29

**Authors:** Aikaterini Dima, Adonis Wazir, Raquel Clark-Castillo, Iordanis Zakopoulos, Shubulade Smith, Fiona Gaughran

**Affiliations:** 1 South London and Maudsley NHS Foundation Trust, London, UK; 2 Institute of Psychiatry Psychology and Neuroscience, London, UK; 3 Surrey and Sussex Healthcare NHS Trust, Redhill, UK; 4 Swansea University, Swansea, UK; 5 University of Texas McGovern Medical School, Houston, USA; 6 Institute of Psychiatry Psychology and Neuroscience, Psychosis Studies, London, UK

**Keywords:** Forensic psychiatry, Length of stay, Public mental health, Forensic services, Mentally disordered offenders

## Abstract

**Background:**

Forensic psychiatry is often associated with long admissions and has a high cost of care. There is little known about factors influencing length of stay (LOS), and no previous systematic review has synthesised the available data. This paper aims to identify factors influencing the LOS in forensic psychiatry hospitals to inform care and interventions that may reduce the length of admissions.

**Methodology:**

A systematic review was conducted by searching major databases, including PubMed, EMBASE and PsycInfo, from inception until May 2022. Observational studies conducted in forensic hospitals that examined associations between variables of interest and LOS were included. Following data extraction, the Newcastle‒Ottawa Scale was used for quality appraisal. No meta-analysis was conducted due to heterogeneity of information; a quantitative measure to assess the strength of evidence was developed and reported.

**Results:**

A total of 28 studies met the inclusion criteria out of 1606 citations. A detailed quantitative synthesis was performed using robust criteria. Having committed homicide/attempted homicide, a criminal legal status with restrictions, and a diagnosis of schizophrenia-spectrum disorders were all associated with longer LOS. Higher Global Assessment of Functioning (GAF) scores were associated with a shorter LOS.

**Conclusion:**

High-quality research examining factors associated with LOS in forensic psychiatry is lacking, and studies are heterogeneous. No modifiable characteristics were identified, and thus, practice recommendations were not made. There is an increasing necessity to understand the factors associated with longer admissions to inform care and increase success in reintegration and rehabilitation. This paper provides recommendations for future research.

**Supplementary Information:**

The online version contains supplementary material available at 10.1186/s12913-024-10863-x.

## Introduction

Forensic psychiatry (FP) has the complex task of caring for mentally disordered offenders: assessing and treating mental illness and simultaneously protecting the public interest where individuals are deemed dangerous [1, 2]. FP has taken on different forms across the world and has developed to different extents [2]. Its scope stretches across several settings: courts, general psychiatric settings, communities, prisons and dedicated forensic psychiatric hospitals. In some places, specialist FP is not available at all [3]. Naturally, FP is heavily intertwined with and dependent on the criminal justice system given its target population, and it is usually up to the justice system to weigh forensic psychiatrists’ expert opinion, decide on criminal responsibility and whether and where a custodial sentence must be served [4].

While the management of forensic patients includes rehabilitation and reintegration into society, protecting the public from potentially dangerous individuals remains an important consideration. Forensic inpatients have lengths of stay (LOS) spanning months or, more commonly, years, increasing in the past decades [5]. These admissions can have LOS that are shorter, equivalent, or sometimes longer than times in detention for imprisoned offenders for the same charge [6]. A high-security bed in the United Kingdom (UK) will be occupied by a forensic service user for an average of 70 months and a medium-security for 26 months [7]. A 2018 survey of 23 medium- and 3 high-security hospitals in England identified 23.5% of inpatients as “long-stayers”, defined as inpatients staying for more than 5 years in medium-security and more than 10 years in high-security services [8]. This phenomenon is not limited to the UK; in Brazil, the average LOS in forensic hospitals was 6 years [9], whereas in the Netherlands, the duration was greater at 8 years [8]. The longer LOS of forensic admissions is also apparent within specific diagnoses. In a 2008 study, patients with schizophrenia spent more days per year hospitalised when they had a forensic admission compared with those who had a non-forensic admission [10].

Forensic mental health services come at a great cost owing to the complexity of care. In the 2019 Scottish government inpatient census, the total number of patients being cared for in a forensic ward in Scotland was 412 [11]. in a population of 5,479,900 people; this highlights the small patient population admitted to such units. According to the Centre for Mental Health Care in the UK, the average cost per annum in England for low-security and medium-security beds is £153,300 and £176,295, respectively, while the cost for high-security beds ranges from £271,560 to £357,335 depending on the specific service [12]. High-security beds in Scotland come at comparable costs, approximately £6195 a week and £322,140 per annum in 2009/10 [13]. Overall, secure care services cost the NHS £821 million in England in 2018/19, corresponding to 10.9% of all public expenditure on mental health services [14]. This high cost is not limited to the UK. In the Netherlands, a forensic bed costs an average of 388 euros per day, adding up to 141,620 euros per patient per year [15]. In Japan, the cost of a forensic bed is US$186,019 per year [16], a cost 4.4 times higher than a non-forensic involuntary admission [17]. This cost, however, must be understood within the broader healthcare landscape and it is essential to recognise the value these services provide. Alternatives such as prolonged high-security imprisonment may incur comparable or even higher expenses, not to mention potentially inferior outcomes in terms of rehabilitation and recidivism rates.

While high cost is one reason to aim for a shorter LOS, the harms of longer admissions must be considered. A lengthy admission can adversely impact patient outcomes and quality of life and reduce the likelihood of future independent living by the individual [18]. Furthermore, it can lead to institutionalisation and social withdrawal [19] and greatly limit autonomy. Forensic environments are by default highly restrictive, with great emphasis placed on security of the individual and others [20], so a longer admission can result in sometimes unnecessary [21] prolonged deprivation of civil liberties for the individuals [22].

However, at present, there is no widely accepted definition of what constitutes a “long LOS” in forensic services; the Butler Committee on Mentally Abnormal Offenders interim report published in 1975 [23] and the Glancy report published in 1973 [24] by the Department of Health and Social Security and the Home Office recommended a maximum 18-month to 2-year stay for medium secure units before an alternative placement is sought. However, these were mere recommendations; research across the UK has repeatedly demonstrated that the LOS in medium- and high-security forensic hospitals often exceeds these numbers [5, 25–27]. It is also worth noting that the original Glancy Report envisaged that *“a significant number of patients are likely to require secure accommodation for longer than 18 months to two years”*. Moreoever, there is no definition of LOS (prolonged or otherwise) in the forensic inpatient setting described in the most recent “Standards for Forensic Mental Health Services” by the Royal College of Psychiatrists and the Forensic Quality Network for Forensic Mental Health Services published in 2019 [28].

Huband et al [29] attempted in 2018 to define what constitutes “long stay” but found that reports were inconsistent across documents and thus it was not possible. They did, however, highlight some characteristics that may contribute to a long stay, such as *“seriousness of index offence, history of psychiatric treatment, cognitive deficit, severity of illness, history of violence, and history of substance misuse”.* This persistent lack of definition of an appropriate LOS from national and international bodies poses significant challenges in research, as investigators have to come up with their own definitions.

In light of the high cost and complexity of care, it is essential to understand what a prolonged admission is and what factors contribute to a longer LOS in FP. The reasoning is dual: from a public health and economic perspective, the financial burden of forensic admissions is heavy and increasing. From a patient-centred care perspective, the potentially detrimental effects of a prolonged admission, albeit being poorly defined, in such restrictive environments must be mitigated. Understanding contributing factors means that services can develop mechanisms to better address prolonged stays and patients’ needs.

While most factors that contribute to LOS are non-modifiable, such as sociodemographic characteristics and forensic or psychiatric history, special consideration must be given to the modifiable factors. Forensic care takes place in a complex system that involves a range of interventions that could influence LOS, such as pharmacological, psychological [30], and occupational therapies [31] or risk management-focused activities.

To date, there has been no systematic review or meta-analysis focusing on the factors influencing LOS as a primary outcome. The main aims of this systematic review were to identify modifiable and non-modifiable factors associated with LOS in forensic psychiatric hospitals and to identify gaps in the relevant literature to formulate recommendations for future research.

## Methodology

A protocol was developed and registered on PROSPERO (ID: CRD42022330535) in May 2022. The systematic review is herein reported according to the Preferred Reporting Items for Systematic Review and Meta-Analysis (PRISMA) checklist [32].

### Search strategy and screening

A systematic search strategy was developed according to the Peer Review of Electronic Search Strategies (PRESS) checklist [33]. The following electronic databases were searched: PubMED, EMBASE and PsycInfo from inception until May 2022, using both subject indexing terms (MeSH, EMTREE) and keywords and their variations. The full strategy is available in Supplementary Material 1.

### Study screening

**Fig. 1:**
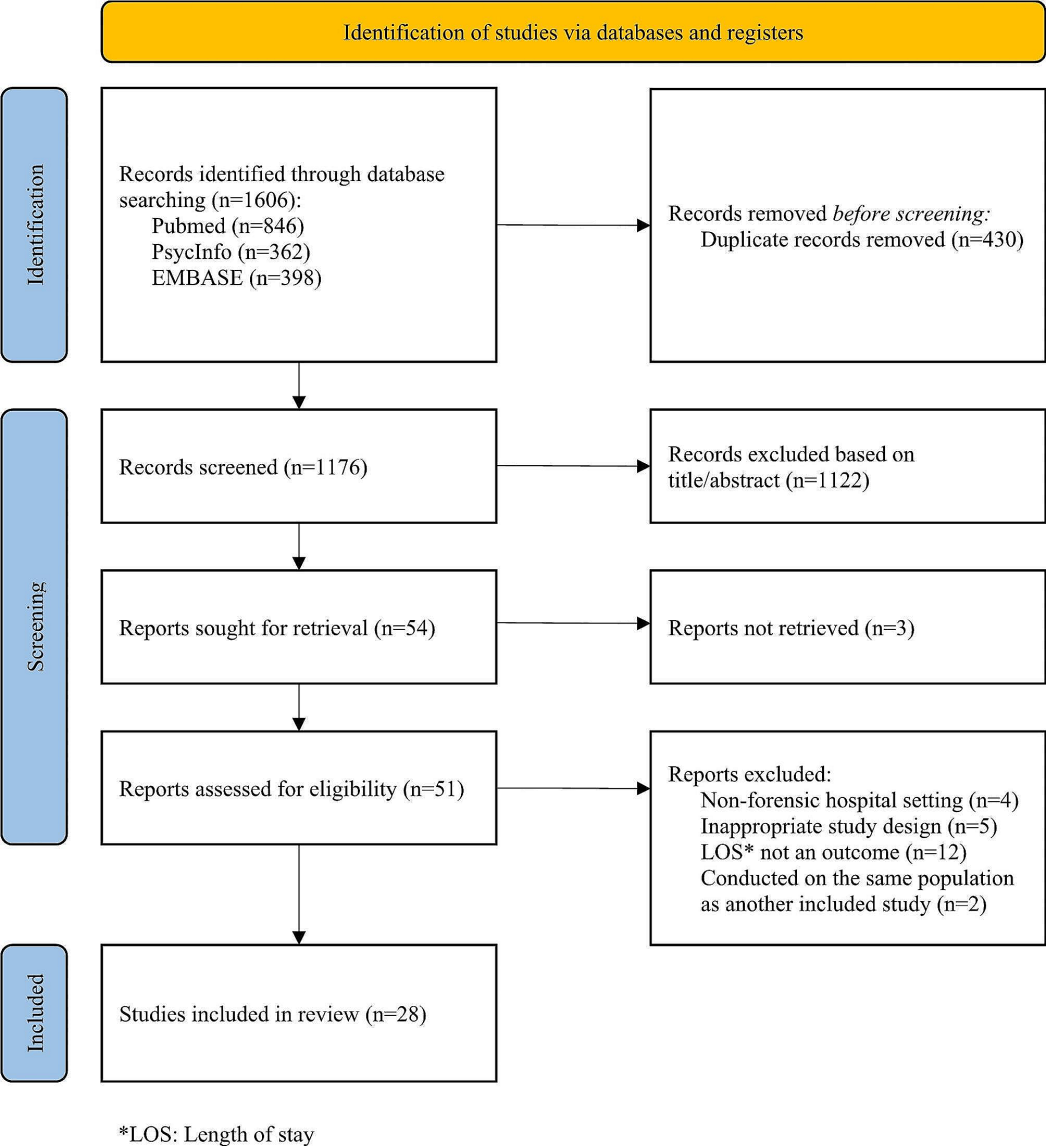
PRISMA flow diagram detailing the identification of articles through databases, numbers of abstracts and reports screened, excluded and included, for this systematic review

Screening of articles was performed by two reviewers (AW and RCC), and any discrepancies were discussed and resolved with the principal reviewer (AD). During the first stage of screening, only titles and abstracts were reviewed to determine eligibility, and a final decision was made at a second stage based on a full-text review of the articles. Any reasons for excluding papers at the full-text review stage were recorded and reported (Fig. 1).

### Inclusion and exclusion criteria

In terms of study design, only observational studies were included due to the nature of the research question. Eligible studies were either prospective or retrospective and used cohort, cross-sectional or case‒control designs. Studies from all countries and jurisdictions were included.

As per the review protocol, studies were included if the patient population examined was adults (aged 18 and above) admitted to forensic psychiatric inpatient settings (defined as specialised forensic hospitals of any security level, including low, medium or high/maximum). Studies conducted in prison, civil or general psychiatric hospitals were ineligible. Studies needed to have reported an association between a variable of interest and LOS to be eligible. LOS could be recorded either as a primary or secondary outcome and measured in any unit of time. LOS was not defined further primarily because the main population of interest was long-stay and difficult-to-discharge patients and secondarily due to a lack of consensus on the definition in forensic psychiatric research.

The variables of interest included sociodemographic characteristics, clinical/psychiatric factors (such as previous diagnoses and treatments), forensic factors (such as characteristics of offence), and legal factors (such as patients’ legal status).

Studies for which full reports were irretrievable and those not reported in English were excluded.

### Quality appraisal

Two authors completed the quality appraisal (IZ and AW), and any discrepancies were discussed and resolved with the principal reviewer (AD). The quality of the included studies was assessed using the Newcastle‒Ottawa Scale (NOS) for case‒control and cohort studies [34] as well as an adapted version of the scale for cross-sectional studies (Supplementary Material 2). The NOS gives an overall score based on three concepts: selection of the sample, comparability of groups or controlling for confounders, and outcomes for an outcome of Good, Fair or Poor.

Research methodologies for LOS studies in health care settings have been extensively examined. There is yet to be an established robust methodology that is recommended, and there is lack of consensus in terms of statistical methods that are most appropriate. Overall, research into factors impacting LOS utilises statistical analyses that compare LOS between two independent samples, which can be done by way of bivariate analysis, regression models, or survival analysis [35]. The critical appraisal was conducted with this consideration in mind, as the NOS allows points for the choice of appropriate statistical tests being reported [34].

### Data extraction

A standardised form for data extraction was developed for the purpose of this review in line with the Strengthening the Reporting of Observational Studies in Epidemiology (STROBE) statement guidance [36]. Data extracted included the year of publication, author details, study design, study period, statistical analysis, study setting, population details, sample size, assessment or follow-up period, source of information, inclusion and exclusion criteria, LOS and variables of interest as explained above. As the outcome variables differed significantly among the included studies, the form was specifically adapted for each one (example provided in Supplementary Material 3). In some studies, the population studied included multiple cohorts, and relevant data were extracted for each cohort separately. All data extraction forms are available upon request.

Data were first extracted on 30/08/2022. Data extraction was performed independently by two reviewers (AW and AD). Any discrepancies were discussed and resolved collaboratively. Authors of included articles were contacted in cases where information and data were missing or inconclusive. Additional data were received only for one study [37].

### Data synthesis

It was not possible to perform a meta-analysis due to heterogeneity among the studies (in methodology and setting) and across selection and coding of variables, lack of data across several studies and the quality of the studies included. Thus, the characteristics of the included studies have been described, including design, setting, sample size, mean age and LOS, and statistics used, which can be seen in Table 1.

**Table 1:** Summary and characteristics of included studies

Study	Author (date)	Country	*N*	Security level	Study Design	Years studied	Duration (years)	Statistical Analysis	Length of stay: mean (SD) in years (unless specified otherwise)	Age, mean (SD)	Female sex (%)
1	Alexander 2011	United Kingdom	138	Medium	Cross-sectional	NS	6	Linear regression	2.8 (median)	30.43(9.274)	21
2	Andreasson 2014	Sweden	127	NS	Cohort	1999–2005	6	Cox regression	2.61	38 (17–79) (median)	19
3	Belfrage 2002	Sweden	150	High (maximum)	Cross-sectional	1997–2001	4	Kruskal Wallis Mann-Whitney U	3.08(3.67)	39 (10)	0
4	Brown 2009	United Kingdom	157	Medium	Cross-sectional	2002–2006	4	Mann-Whitney U	1.97 (median)	38.9 (Restricted) 35.1 (Civil/Prison)	0
5	Chester 2018	United Kingdom	402	Medium and High	Cross-sectional	2013	NA	Mann-Whitney U	12.67(8.2) (ID) 14.95(8.78) (nID)	40 (ID) 45 (nID)	14.2
6	Colwell 2011	United States	71	High (maximum)	Cross-sectional	NS	NS	Logistic regression	0.32(0.21)	37.9 (11)	0
7	Davoren 2015	Ireland	287	Medium	Cohort	2010–2014	4	Cox regression	0.83(0.10^) (Males) 0.56(0.16^) (Females)	34.9(10.5)	16.7
8	Dell 1987	United Kingdom	187	High	Cross-sectional	1972–1984	12	Chi-squared	NS	NS	0
9	Duke 2018	United Kingdom	2287	Medium and High	Cross-sectional	2013	NA	Logistic regression	11.75(5.75,16)* (HS/LS) 3.67(1.75,5.92)* (HS/nLS) 4.25(1.67,6.5)* (MS/LS) 1.17(0.5,2.33)* (MS/nLS)	45.43(9.67) (HS/LS) 36.15(9.72) (HS/nLS) 43.87(11.74) (MS/LS) 34.68(11.21) (MS/nLS)	14.3
10	Eckert 2017	Netherlands	139	High	Cross-sectional	2006–2013	7	Logistic regression	10.58(4.91) (LFPC) 8.32(4.69) (RFPC)	52.97(8.09) (LFPC) 44.01(10.97) (RFPC)	0
11	Edwards 2002	United Kingdom	225	Medium	Cohort	1983–1996	13	Linear regression Chi-squared	2.17	NS	14.7
12	Esan 2015	United Kingdom	138	NS	Cross-sectional	NS	6	Mann-Whitney U	3.62(3.02) (D/ASD) 3.76(2.69) (D/nASD) 4.18(4.30) (nD/ASD) 4.88(4.12) (nD/nASD)	30.14(9.14) (ASD) 30.56(9.38) (No ASD)	21
13	Gosek 2020	Poland	150	Medium	Cross-sectional	2014–2018	4	Mann-Whitney U Kruskal-Wallis Linear regression	3.26(3.54)	40.07(12.99)	18
14	Green 1998	Australia	586	NS	Cross-sectional	1989–1995	6	Cox regression	0.40(0.75) (Males) 0.41(0.49) (Females)	30.6(10.2)	11.7
15	Griffiths 2018	United Kingdom	347	Low and Medium	Cross-sectional	2007–2015	8	*t*-test	2.56(1.65) (Non-secluded) 2.79(1.90) (Secluded)	35.2(12.8) (Non-secluded) 30.8(10.7) (Secluded)	35
16	Hillbrand 1996	United States	53	High (maximum)	Cross-sectional	1989–1990	1	*t*-test	0.93(1.44) (ISIB) 2.34(1.23) (RSIB)	30.0 (ISIB) 37.4 (RSIB)	0
17	Long 2012	United Kingdom	70	Medium	Cross-sectional	2002–2010	8	Chi-squared Log-linear analysis	1.63(0.69)	30.4(7.6)	100
18	McKenna 2019	United Kingdom	194	High	Cross-sectional	2017	NA	Mann-Whitney U	5.54	37.35	0
19	Messina 2011	United States	1915	High (maximum)	Cross-sectional	2003–2010	7	Cox regression	0.53	40	NS
20	Moran 1999	United States	101	High (maximum)	Cross-sectional	NS	5	Linear regression	NS	32.42(9.8) (Males) 36.14(9.4) (Females)	13.9
21	Moulden 2020	Canada	14	NS	Cross-sectional	2009–2011	2	NS	3.24	36.36(12.47)	35.7
22	Pàv 2022	Czechia	260	NS	Cross-sectional	2015–2020	5	Linear regression	1.36(1.91)	42.26(12.79)**	12.7
23	Rodenhauser 1988	United States	376	High (maximum)	Cross-sectional	1980–1984	4	ANOVA	0.42(0.45)	NS	NS
24	Ross 2012	Germany	899	NS	Cross-sectional	NS	NS	*t*-test Chi-squared Mann-Whitney U	5.77(5.61)	NS	26.6
25	Shah 2011	United Kingdom	259	Medium	Cross-sectional	1999–2008	10	Logistic regression	2.05(2.94)	30.9(8.6)	9.7
26	Smith 2004	United Kingdom	34	Low	Cohort	1991–2003	12	Linear regression	5.5(5.8)	40.5 (Special) 36.8 (Control)	0
27	Verstegen 2017	Netherlands	503	NS	Cross-sectional	2008–2014	6 years	*t*-test	1.42(1.40) (Violent group) 0.67(0.88) (Non-violent group)	37.2(10.2)	19.3
28	Wint 1994	United Kingdom	43	High	Case-control	NS	NS	Chi-squared	NS	29.9(8.73) (long stay group) 30.26(6.98) (short stay)	0

NS = Not specified

NA = Not applicable

ID/nID = Intellectual disability/No intellectual disability

HS = High security

ASD = Autism Spectrum Disorder

MS = Medium security

LS/nLS = Long-stayers/Non-long-stayers

LFPC = Long-term Forensic Psychiatric Centre

RFPC = Regular Forensic Psychiatric Centre

^ Standard Error

*Median (P25, P75)

**At discharge

First, all variables identified during data extraction across all studies (380) were collected. These were then grouped and pooled into matching categories (e.g., diagnosis of paraphilias and diagnosis of sexual deviance under the category of sexual preference disorders, as per ICD-10, the diagnosis system in place at the time of data collection). To accurately group the different variables, each article was revisited, and their specific definitions were recorded. Where variables were not clearly defined, decisions were made based on clinical judgement when possible, or the variables were excluded from the grouping. All variables that were examined by only one study were excluded. For each of the remaining variables, the number of studies that investigated them, as well as the number of studies that showed positive, negative or non-significant correlations with the LOS, were reported, stratified by the quality of the respective study (Table 2). A list of variables studied per article can be found in Supplementary Material 4.

**Table 2:** Factors examined in at least two studies

Variables	*N*	Good +	Good -	Good NS	Fair +	Fair -	Fair NS	Poor +	Poor -	Poor NS
**Demographics**										
Age	16	1		5	1		4			5
Female sex	8		1	3		1				3
Male sex	9	1		2	1		1			4
White ethnicity	5	1		2						2
Afro-caribbean/African-american ethnicity	4			2					1	1
Asian ethnicity	3			2						1
Mixed ethnic background	2			1						1
Immigrant	2			1		1				
**Education/Employment**										
No or special primary education	2						1			1
Any level of education	5				1	1	2			1
No employment history	4				1		1			2
Any employment history	8					2	3		1	2
**Family Status**										
Married/engaged	4			1		2				1
Single or in unstable relationship	4				1		1			2
Widowed	2						1			1
Divorced	2					1				1
Parent	2		1				1			
**Admission Source**										
Community	2	1		1						
High-security hospital	2	1		1						
Medium-secure unit	2			1	1					
Prison or court	2	1		1						
**Diagnosis**										
Adjustment disorder	2		1							1
Affective disorders	6	1					2		1	2
ASD^1^ / Pervasive developmental disorders	4						2			2
Anxiety disorders	4			1			1			2
Bipolar	3			1					1	1
Depression	2									2
Impulse control disorder	2			1						1
Intellectual or Learning Disability	4		1		1					2
Organic mental disorders	4						1			3
Personality disorders	9			2		1	2		2	2
Personality disorders– Cluster B	2				1					1
Sexual preference disorder	4				1		2			1
Schizophrenia-spectrum disorder	6	1		1	2			1		1
Schizophrenia	3			2				1		
Schizoaffective Disorder	2			1				1		
Substance use disorders	9					2	3			4
Substance-induced Psychosis	2			1						1
Psychosis-related	11	1		1	2	1	1	1		4
**Scores**										
IQ^2^ score	3						2		1	
GAF^3^ Score	3		2						1	
DUNDRUM-1^4^ Total Score	2	1			1					
HCR20^5^ Score– Clinical	3			1	1		1			
HCR20 Score– Historical	3			1			2			
HCR20 Score– Risk	3			1	2					
HCR20 Score– Total	4			2	1		1			
**Forensic History**										
Age at first conviction	4			2			1			1
Any previous convictions	3									3
Number of previous convictions/sentences/incarcerations	3			2						1
Presence of previous major offence	4			1			1			2
History of previous incompetencies to stand trial	2									2
Amount of previous forensic treatment	3	1								2
**Psychiatric History**										
Previous psychiatric treatment	7	1		1			1	2		2
Duration of mental illness in years	2			1				1		
Number of previous psychiatric admissions	5			1			1	1		2
**Other history**										
Family history of mental illness	2						1			1
Any past history of abuse	4				1					3
**Index Offence**										
Age at index offence	2						1		1	
Index offence of sexual nature	9	1		2	1		1	1		3
Index offence of homicide or attempted homicide	8	1		1	2			1		3
Major index offence	20	3		2	3	1	2	3	2	4
Minor index offence	8			2		2	1			3
Nil offence	3			2			1			
Index offence under influence of alcohol/psychoactive substances	2					1				1
**Institutional Aggression**										
Violence to others	4			2				1		1
Number of acts of violence	2			1					1	
Presence of violence to self	2			2						
Amount of absconding	2			1	1					
Seclusion and/or restraint	5			1			1	1		2
**Legal Category/Status**										
Legal category– Mental Illness	3			1	1					1
Legal category– Psychopathic Disorder	3			1		1				1
Legal status– civil	6		1	2			1			2
Legal status– prison transfer	5		1	1			1			2
Legal status– criminal section	5			1			1	1		2
Legal status– criminal section with restrictions	7	1		1	2			1		2
Legal status– no criminal responsibility	4			2		1				1
Legal status– remand order	3	1		1						1
**Treatment**										
Psychotherapy engagement	2					1				1
Attendance of groups while admitted	2		1							1

^1^ ASD: Autism Spectrum Disorder

^2^ IQ: Intellectual Quotient

^3^ GAF: Global Assessment of Functioning

^4^ DUNDRUM-1: Dangerousness, Understanding, Recovery and Urgency Manual - Triage Security Items (96)

^5^ HCR20: Historical, Clinical and Risk Management

To identify factors of interest despite heterogeneity, the reviewers applied the same methodology as Luppa et al [38] and Moore et al [39]. To assess the quality of the evidence for each of the variables, a judgement was made based on three criteria: (a) the number of studies that examined a particular variable, (b) the quality of those studies, and (c) the consistency of the results across studies. Variables that did not meet the specified criteria or those that were evaluated by fewer than 3 studies were determined to have “inconclusive evidence”. Evidence was further deemed “inconclusive” when there was an equal sum of findings pointing in different directions. A summary of the quality of evidence can be found in Table 3, as per the following criteria:

- “Strong” quality of evidence for a particular variable was considered if it was examined in at least 3 good quality studies, and the results were similar (following the same direction of association) in at least 75% of the studies that looked at it.
- “Moderate” was based on having consistent findings in at least 50% of studies across a minimum of 2 good quality studies.
- “Weak” was attributed to variables that were examined by at least 3 studies, 1 of which was assessed to be of good quality, with similar results in at least 50%, or similar results in at least 4 studies classed as either fair or poor quality.

**Table 3:** Quality of evidence for variables with conclusive results

Variables	Quality of evidence	Conclusion
Age	Strong	No significant correlation with length of stay
Female sex	Strong	No significant correlation with length of stay
Male sex	Strong	No significant correlation with length of stay
White ethnicity	Strong	No significant correlation with length of stay
Legal status– civil	Strong	No significant correlation with length of stay
African-Caribbean/African American ethnicity	Moderate	No significant correlation with length of stay
Asian ethnicity	Moderate	No significant correlation with length of stay
Personality disorders diagnosis	Moderate	No significant correlation with length of stay
Schizophrenia-spectrum disorders	Moderate	**Positive correlation with length of stay**
Schizophrenia diagnosis	Moderate	No significant correlation with length of stay
GAF^1^ Score	Moderate	**Negative correlation with length of stay**
HCR20^2^ Score– Total	Moderate	No significant correlation with length of stay
Age at first conviction	Moderate	No significant correlation with length of stay
Number of previous convictions/sentences/incarcerations	Moderate	No significant correlation with length of stay
Index offence of sexual nature	Moderate	No significant correlation with length of stay
Index offence of homicide or attempted homicide	Moderate	**Positive correlation with length of stay**
Minor index offence	Moderate	No significant correlation with length of stay
Nil offence	Moderate	No significant correlation with length of stay
Violence to others while institutionalized	Moderate	No significant correlation with length of stay
Presence of violence to self while institutionalized	Moderate	No significant correlation with length of stay
Legal status– prison transfer	Moderate	No significant correlation with length of stay
Legal status– criminal with restrictions	Moderate	**Positive correlation with length of stay**
Legal status– no criminal responsibility	Moderate	No significant correlation with length of stay
Previous psychiatric treatment	Moderate	No significant correlation with length of stay
Diagnosis of substance use disorder	Weak	No significant correlation with length of stay
Diagnosis of affective disorders	Weak	No significant correlation with length of stay
ASD^3^ / Pervasive developmental disorders diagnosis	Weak	No significant correlation with length of stay
Diagnosis of anxiety disorders	Weak	No significant correlation with length of stay
Diagnosis of organic mental disorders	Weak	No significant correlation with length of stay
Psychosis-related diagnoses	Weak	No significant correlation with length of stay
Presence of previous major offence	Weak	No significant correlation with length of stay
HCR20 Score– Historical	Weak	No significant correlation with length of stay
Legal status– criminal section	Weak	No significant correlation with length of stay
Number of previous psychiatric admissions	Weak	No significant correlation with length of stay
Seclusion and/or restraint while institutionalized	Weak	No significant correlation with length of stay

^1^ GAF: Global Assessment of Functioning

^2^ HCR20: Historical, Clinical and Risk Management

^3^ ASD: Autism Spectrum Disorder

## Results

The search yielded a total of 1606 records across three databases. After de-duplication, 1176 records were left for screening. Following the title and abstract screening stage, 1122 records were excluded, and 54 records were eligible for full text review. Reasons for excluding full papers were recorded and reported (Fig. 1). Quality appraisal and data extraction were completed for 28 studies in total.

The list of included studies and their characteristics are presented in Table 1. In terms of study design, cross-sectional methodologies were largely overrepresented (23 studies), whereas fewer prospective or retrospective cohort [40–43] and case‒control designs [44] were identified.

A total of 13 studies were conducted in the UK [42–54], five studies in the USA [55–59] and two studies in Sweden [40, 60] and the Netherlands [61, 62]. The rest of the studies were conducted in Australia [63], Canada [64], Czechia [37], Germany [65], Ireland [41], and Poland [66]. The total sample size across all studies was 10,112, with the smallest being 14 [64] and the largest being 2287 participants [52].

The level of hospital security was not specified or applicable in seven studies [37, 40, 53, 61, 63–65]. There were three studies that were conducted across more than one level of security (2 studies in medium- and high-security hospitals [50, 52] and one study in low- and medium-security hospitals [54]). The rest of the studies were conducted across only one level of security, with 10 studies conducted in maximum- or high-security hospitals [44, 45, 51, 55–60, 62], seven studies in medium-security hospitals [41, 42, 46–49, 66] and one study in low-security hospitals [43].

Most studies reported mean ages of their samples, which ranged from 29.9 years [44] to 52.97 years [62]. The age of participants was not reported in four studies [42, 51, 59, 65]. The majority of studies included predominantly males, with percentages ranging from 64.3% [64] to 90.3% [46] of all participants. Nine studies [43–45, 49, 51, 55, 56, 60, 62] included only male participants. One study examined female patients only [47]. One study only looked at forensic patients with a learning disability [48].

The overall LOS was reported in most studies, but others reported means of the groups without giving an overall picture. LOS ranged from 0.32 years (equivalent to 116.9 days) in a study conducted in a maximum-security hospital in the USA [55] to 14.95 years in a study conducted in both medium- and high-security settings in the UK [50]. In the latter study, there was no differentiation in the reporting between the medium and high secure settings in terms of LOS.

### Quality of studies

Following critical appraisal, seven studies were judged to be of good quality, nine studies of fair quality and the rest of the studies were poor. The most common issues identified were a lack of comparators or adequate control for confounders and offering no justification for sample size selection. Strong indicators across studies commonly included random selection and representativeness of the sample. Moreover, most investigators had direct access to secure and up-to-date medical records but did not account for reporting bias by the authors of the records. The outcomes of the studies were reported to varied extents, with a minority of the studies only including statistically significant results in their manuscripts. Details of the critical appraisal can be found in Table 4.

**Table 4:** Critical appraisal of included articles

Author (date)	Study	Study Design	Selection	Comparability	Outcome	Quality
Andreasson 2014	2	Cohort	3	1	3	Good
Chester 2018	5	Cross Sectional	3	2	3	Good
Davoren 2015	7	Cohort	3	1	2	Good
Duke 2018	9	Cross Sectional	3	1	3	Good
Edwards 2002	11	Cohort	3	1	3	Good
Messina 2011	19	Cross Sectional	3	1	3	Good
Smith 2004	26	Cohort	3	1	3	Good
Belfrage 2002	3	Cross Sectional	2	2	3	Fair
Brown 2009	4	Cross Sectional	2	1	3	Fair
Eckert 2017	10	Cross Sectional	2	2	3	Fair
Green 1998	14	Cross Sectional	2	1	3	Fair
Griffiths 2018	15	Cross Sectional	2	2	3	Fair
Long 2012	17	Cross Sectional	1	0	3	Fair
Pav 2022	22	Cross Sectional	2	1	3	Fair
Ross 2012	24	Cross Sectional	2	2	3	Fair
Wint 1994	28	Case-control	2	1	3	Fair
Alexander 2011	1	Cross Sectional	2	0	3	Poor
Colwell 2011	6	Cross Sectional	1	0	3	Poor
Dell 1987	8	Cross Sectional	2	0	3	Poor
Esan 2015	12	Cross Sectional	1	2	3	Poor
Gosek 2020	13	Cross Sectional	2	0	3	Poor
Hillbrand 1996	16	Cross Sectional	2	0	3	Poor
McKenna 2019	18	Cross Sectional	2	0	3	Poor
Moran 1999	20	Cross Sectional	2	0	3	Poor
Moulden 2020	21	Cross Sectional	1	0	1	Poor
Rodenhauser 1988	23	Cross Sectional	2	0	3	Poor
Shah 2011	25	Cross Sectional	2	0	3	Poor
Verstegen 2017	27	Cross Sectional	2	0	3	Poor

### Studied variables

Overall, a total of 380 variables were examined. Variables that were similar were pooled and grouped. Table 2 shows all 79 factors that were assessed by at least two articles. Although the broad categories of variables were predetermined, the ones displayed are a result of what was identified in the review.

The most studied factor was the presence of a major index offence (defined as homicide, attempted homicide, serious bodily assault, armed robbery, kidnapping and arson), which was examined in 20 articles. This was followed by age (16 articles), psychosis-related diagnoses (excluding schizophrenia) (11 articles), male sex (nine articles), and history of substance use disorder (nine articles).

Some noteworthy variables that were examined by a single study and were not included in the discussion were treatment with clozapine [66], treatment with > 1 antipsychotic [66], persistent psychotic symptoms over the past 6 months of admission [66], age at first psychiatric admission [65], age at the onset of psychiatric symptoms [43], and substance abuse during admission [40].

### Predictors of length of stay

The present review demonstrated varying quality of evidence for the different variables studied (Table 3). Most variables had no significant correlation with LOS. Significant correlations could be made for some factors. A lower GAF score (moderate evidence), an index offence of homicide or attempted homicide (moderate evidence), a legal status of criminal section with restrictions (moderate), or a diagnosis of schizophrenia-spectrum disorders (weak evidence) were correlated with a longer LOS.

## Discussion

The primary aim of this systematic review was to identify factors that influence LOS in forensic inpatient settings and synthesise findings across high-quality studies. At the time of this study, no previous systematic review has focused entirely on the factors that influence LOS in forensic settings. A systematic review in 2015 examined factors influencing key forensic outcomes [67]; however, this review included fewer articles and was not focused on LOS. The authors only extracted statistically significant results, and there was no data synthesis. There was no meta-analysis for reasons similar to those reported above. Similarly, a rapid review conducted in 2018 by Huband et al [29] attempted to answer a set of different questions, including what constitutes a long stay, what are the characteristics of long-stay patients and what factors predict the LOS. This review utilised a rapid rather than a systematic review methodology and more restrictive inclusion criteria, such as factors that were only explored by multivariate analysis. Although this may control for confounding, it nevertheless excludes other robust statistical analyses. Most importantly, however, both Huband et al. and Sedgwick et al. described their findings narratively and did not conduct a data synthesis.

### Main findings

While no meta-analysis was performed, we identified factors of interest that are supported by strong, moderate or weak quality of evidence, as explained above. The primary reason for identifying relevant variables remains to inform better care and discharge planning for forensic patients and allow for targeted treatment and distribution of health services; however, for most of the studies, no link to clinical practice was made.

One of the aims of this review was to identify modifiable factors for the purpose of directing potential future interventions. However, most factors with good quality evidence were non-modifiable. While most studies looked at historical information (e.g., psychiatric history, forensic history), few examined characteristics of treatment or institutional behaviour, and no studies looked into details of daily functioning with the exception of GAF scores. Some clinically interesting variables included refusal of treatment [59] and involuntary treatment administration [59], treatment with more than one antipsychotic [66] and having treatment-resistant psychosis [37], i.e., non-responsive to at least two different antipsychotics.

Overall, it appears that none of the sociodemographic variables appeared to be associated with LOS, including age, sex, ethnicity, employment, and family status, supported by strong and moderate quality of evidence. This differs from general psychiatric wards, where African-Caribbean patients appear to experience prolonged stays [68] in the UK. A common difficulty in research remains the consistent measurement of these variables, as while frequently included in studies, authors use different terminology. We explore this complexity in more detail below.

Interestingly, civil legal status was not associated with longer admissions. This finding, however, is limited to psychiatric services in the UK, where the term refers to an involuntary admission under Part II of the Mental Health Act (MHA) 1983 [69] (amended in 2007 [70]) either for assessment or treatment. A criminal (or forensic) section refers to an involuntary admission under Part III of the MHA, which may include court-imposed restrictions, as indicated previously in this paper. Admissions under civil detention are largely into general psychiatric hospitals, but these patients can be found in forensic wards at varying degrees [71] and are transferred usually as a result of increased risk and behavioural disturbance that cannot be managed on general psychiatric wards.

The initial expectation was that civil detention would correlate with shorter LOS due to the absence of an index offence and judiciary involvement. However, on closer inspection, these patients appear to pose greater management challenges, with more frequent episodes of aggression [72, 73]. This may nullify the positive effect of not having committed an offence.

Similarly, an absence of index offence and institutional aggression during hospitalisation (including the requirement of seclusion and restraint) did not influence LOS. Institutional aggression was quantified by the number of incidents perpetrated by the individual throughout their admission. The common expectation in forensic psychiatric settings is that heightened risk would result in a prolonged stay to protect either the individual or society– in some cases, both. There is, however, a need for further investigation to corroborate these findings.

Conversely, and unsurprisingly, patients in the UK admitted under a criminal section with restrictions tended to stay in the hospital for longer periods of time. A criminal section implies that an individual has been convicted of a crime by court but is identified to have a mental disorder and is in need of medical assessment and/or treatment. A criminal section with restrictions means that the individual cannot be granted leave or be discharged without prior approval by the Ministry of Justice and is usually reserved for more serious offences.

The most examined variable across all studies was a major index offence. Unlike the findings of Sedgwick et al [67] and Huband et al [29], the evidence suggesting that having committed a broadly defined major offence prolongs the LOS was inconclusive, primarily due to the inconsistency of results across studies. A major index offence is associated with an increased risk of violence and more conservative discharge planning. Nonetheless, it is important to note that despite being inconclusive, there were nine studies [40, 42, 47, 51, 55, 59, 63, 65, 66] where having committed a major index offence was reported as a predictor of longer LOS, and there is a need for more high-quality studies to decipher this relationship. On the other hand, having committed specifically homicide or attempted to commit homicide was associated with an increase in LOS.

With regard to rating scales, GAF was the only scale to have an association with LOS, and this was negative. This finding appears logical, as a higher GAF score is associated with fewer symptoms and better social and occupational functioning [74]. GAF has also been found to have a strong predictive validity of one-year treatment outcomes [75] and thus appears to be a reliable tool to monitor progress and recovery.

In terms of diagnosis, while schizophrenia-spectrum disorders (SSD) were linked to prolonged LOS, the quality of evidence was moderate and did not extend to the specific diagnosis of schizophrenia or other psychotic disorders. This may be due to limited high-quality studies and sample sizes, but it could also be partially explained by the relative dearth of evidence for optimal management of SSD such as schizophrenia [76] and the widespread recognition of the burden of such a diagnosis [77]. One key aspect of the impact of diagnosis on LOS, however, would be the existence of comorbidities, such as substance use disorder, which has a very high prevalence among patients with SSD [78]. This association should be the focus of future research.

One key finding across this review was a consistently higher LOS among patients in the UK, particularly in comparison to the USA. The importance of different organisations and the provision of forensic psychiatry cannot be understated. However, at least when comparing services of similar nature (i.e., high-security settings), such a stark difference could be explained by several factors. In US secure hospitals, the focus is on competency restoration to stand trial, rather than long-term treatment. If competency cannot be restored, then charges must be dropped, and the individuals are either released or admitted under a civil Sect. [79]. There has been a recent drive to reduce the prison population and a focus on community services in the USA, which may lead to faster movement between services [80, 81]. Additionally, the influence of a public, nationalised health system in the UK might also be relevant, as is the court diversion system [82]. Very few studies from other countries were included, and thus, no inferences could be made.

### Variation of definitions

One striking finding of this review was the often-extreme variations in definitions of relevant variables. Some of these variations, such as the level of security or lack thereof, were expected, as FP is structured differently across the world and is dependent on local legislation [83].

This variation included the primary outcome variable, LOS, defined mostly as either LOS at the time of the study or LOS until discharge. As there is no accepted threshold of a “lengthy” inpatient stay, authors have had to devise their own definition which differ across studies. A total of 19 out of 28 studies did not define LOS at all and rather drew comparisons based on the LOS of the included patients at a specific point in time. The most common cut-off point used was the 2-year mark [42, 44, 46, 51, 60, 65]. Chester et al [50] and Duke et al [52] defined a prolonged LOS as more than 5 years in medium secure care and 10 years in high secure care and compared patients in these groups with those who did not meet the criteria. For Alexander et al [48], the difficult-to-discharge group was determined based on the median LOS of the patients who had been discharged at the time of the study. The need for a universal definition remains a very important point for consideration in forensic research.

The definition of ethnicity also poses a challenge, as there is no universally agreed system of classification, and such a classification would largely depend on local context [84]. The relationship between ethnicity and outcomes may be affected by minority status, which is not constant globally. In our review, different studies used different ways of reporting or had much broader categorisations, and it is possible that there may be associations missed due to the difficulties in grouping.

The definition and measurement of age was surprisingly varied. The definitions encountered in the included articles were age on admission, age on discharge, and age during the study. In terms of association with LOS, these varying definitions represent different variables and should be studied further to corroborate the accuracy of the conclusion itself.

Another important finding to highlight was the lack of effect size measurement and differences in statistical analysis and reporting across multiple studies. *P* values alone are not sufficient to identify an association, and a statistically significant result is much more likely with larger sample sizes [85]. Effect sizes are thus necessary to understand the extent of the difference between the groups and provide an added layer of security that the result is not only statistically but also clinically significant.

### Limitations

This review aimed to answer a complex question. The authors focused on synthesising evidence across different countries, cultures and economic systems, all factors that are imperative in forensic psychiatry. There is thus an inherent weakness in any similar quantitative‒qualitative analysis and one that is unlikely to be resolved in the future.

As with all systematic reviews, and despite best efforts to include all relevant terms and keywords in the search strategies, it is possible that relevant and high-quality studies might have been missed as non-English articles were excluded and the search was limited to three databases. However, the search strategy was initially piloted across all key databases, and those with relevant results were included in the review.

As the present review focused on observational studies, the evidence identified carries several of the limitations associated with this design. Cross-sectional designs in particular—which were overrepresented—have limited capacity to assess causal relationships between LOS and variables of interest. It is, however, a cost-effective and easy way to look at a snapshot of information, and it is preferred where access to detailed medical records is readily available.

Most commonly, forensic mental health services include 3 levels of security. This is the case for the UK [5], Sweden [86], Poland [87], Canada [88], Germany [89] and Australia [90]. Dutch forensic services provide four levels of security, determined by the patient’s legal and clinical status [8]. In some jurisdictions, all three levels are provided on the same site. However, even where the level of security appears similar, service provision and expectations may not be comparable, as demonstrated by the stark LOS difference among high-secure hospitals in the US and UK. It would be important for future research to focus on specific levels of security that are aligned both in terms of risk stratification and scope of practice, particularly when conducting international reviews.

A key limitation in both the available evidence and this study is highlighted by the lack of a meta-analysis. Studies looking at LOS in forensic settings are extremely heterogeneous in terminology, measurement of variables, statistical analysis, measurement of effect sizes, and even in reporting of results. Even beyond the heterogeneity, most of the included studies explored a large number of variables but without having a prior hypothesis and without reporting effect sizes. This is not the case only in FP research but also in general psychiatry and was highlighted by a 2011 review on the LOS in general psychiatric inpatients in the USA [91]. It is the authors’ hope that this review can bring this issue forward and encourage future authors to follow a list of recommendations that have been compiled and can be found below.

Lastly, an important observation was data dredging. Data dredging increases the chance of identifying possible associations, particularly statistically significant ones, through introducing multiple variables or multiple categorisations of variables [92, 93]. Across several studies in this review, the list of variables was lengthy, and among the statistically significant associations identified, some often lacked practical or clinical significance and had poor generalisability outside the study population. Such variables could include inappropriately grouped diagnoses (e.g. intellectual disability and dementia) or extensive categorisation of demographic history (e.g. employment or social history).

### Implications for future research and practice

The present body of work adds significant value to the literature, if only for the gaps that have been identified and described at length. A set of variables, including having committed homicide/attempted homicide, a criminal status with restrictions and schizophrenia-spectrum disorders, were found to have evidence of varying quality to suggest that they may prolong LOS. These findings should be re-examined using higher quality research to prove this association and understand how care can be adapted to account for them– perhaps through the development of different pathways for rehabilitation, according to the details of the index offence (aside the levels of security). On the other hand, the importance of GAF scores has been highlighted, as they are negatively associated with LOS and can be a quick and efficient tool to use in daily practice. While the evidence is moderate, it implies that both severity of illness and daily functioning are important aspects to consider across care planning, and future researchers are encouraged to use them as guidance for examining response to rehabilitation.

Regarding heterogeneity, unless a set of commonly examined variables are standardised, it is unlikely that a meta-analysis will be possible in the future. A set has been compiled below, and the authors encourage investigators to consider this in their practice. It is also crucial that modifiable variables, particularly in terms of treatment (pharmacological and non-pharmacological) characteristics, are explored. While the evidence for psychotherapy treatment was inconclusive due to a lack of high-quality studies and consistency, Long et al [47] identified that engagement reduced LOS, while Moulden et al [64] reported a reduction in LOS for patients receiving Dialectical Behaviour Therapy (DBT), albeit in a very small sample. Another non-pharmacological treatment option that ought to be explored is occupational therapy, as it plays a significant role in recovery [94]: Messina [57] highlighted a shorter LOS among those with higher attendance at therapeutic groups while in the hospital, and there was evidence in this review to suggest that the same applies to higher GAF scores [40, 55, 57], which are partly based on psychosocial and daily functioning.

## Conclusion and recommendations

This systematic review was conducted to explore factors associated with LOS for patients in forensic psychiatry units. A limited number of studies with adequate quality to make conclusions was identified, but it was possible to identify factors with moderate or weak evidence to support their correlation (or lack thereof) with LOS. There is moderate to weak evidence of a positive effect among having committed homicide/attempted homicide, a criminal section with restrictions and schizophrenia-spectrum disorders and LOS. Moderate evidence suggests a negative correlation between the GAF score and LOS. There are varying levels of quality of evidence that suggest no significant correlation between the other variables that were reviewed and LOS.

This review has highlighted gaps and inconsistencies in current research, as well as heterogeneities in methodology, definitions, and analyses. Thus, some recommendations for researchers to consider in the future have been compiled:

I. In terms of methodology, prospective cohort designs and examining specific sets of variables from admission (including pathway to admission) onwards will provide a more robust methodological approach, allowing inferences on any associations with LOS. Moreover, due to forensic services relying mostly on involuntary admissions, loss to follow-up is highly unlikely.
II. Use standardised definitions for variables including age, sex, employment status, marital status, ethnicity, race, migration, or citizenship status where possible. This endeavour becomes more complex across borders, and the authors recommend using standardised tools such as census questionnaires.
III. If there is grouping of diagnoses, provide additional data on each diagnosis made using consistent classification systems, such as ICD-11, to facilitate later meta-analyses. Additionally, any comorbidities (historic or current) need to be clearly mentioned and their associations examined.
IV. Collect treatment-related variables such as medication choice, polypharmacy, engagement in psychotherapy, and engagement in occupational therapy.
V. Examine variables related to presentation as an inpatient, including behavioural disturbance and institutional aggression, ongoing symptoms of mental illness, and use of substances.
VI. Future research should focus on modifiable characteristics (such as recommendations IV and V) while continuing to collect non-modifiable characteristics in a uniform manner.
VII. Avoid overinclusion of variables, particularly where clinical significance may be questionable.
VIII. Report on effect sizes where a statistically significant result has been identified and present this finding in a clear and concise manner.

### Electronic supplementary material

Below is the link to the electronic supplementary material.

Supplementary Material 1

Supplementary Material 2

Supplementary Material 3

Supplementary Material 4

## Data Availability

Additional data available upon request.

## Abbreviations

LOS: Length of stay
GAF: Global Assessment of Functioning
FP: Forensic Psychiatry
UK: United Kingdom
PRISMA: Preferred Reporting Items for Systematic Review and Meta-Analysis
NOS: Newcastle‒Ottawa Scale
STROBE: Strengthening the Reporting of Observational Studies in Epidemiology
SSD: Schizophrenia-spectrum disorder
DBT: Dialectical-Behavioural Therapy
NS: Not specified
NA: Not applicable
ID/nID: Intellectual disability/No intellectual disability
HS: High security
ASD: Autism Spectrum Disorder
MS: Medium security
LS/nLS: Long-stayers/Non-long-stayers
LFPC: Long-term Forensic Psychiatric Centre
RFPC: Regular Forensic Psychiatric Centre
