# Supplementary material for: Factors influencing the length of stay in forensic psychiatric settings: a systematic review
Source: BMC Health Serv Res. 2024 Mar 29;24:400. doi: 10.1186/s12913-024-10863-x (PMC10981349; doi:10.1186/s12913-024-10863-x)
Supplement: Supplementary file 1 — Supplementary Material 1 [file 12913_2024_10863_MOESM1_ESM.docx]

## **Supplementary Material 1: Search Strategy**

**PubMED:**

1 forensic psychiatry[MeSH Terms]

2 forensic psychiatr*

3 (secure OR forensic) adj3 (unit OR ward OR hospital OR department OR service OR team)

4 mentally disordered offender

5 length of stay[MeSH Terms]

6 length of stay

7 stay length

8 length of admission

9 admission length

10 hospital stay

11 duration of stay

12 duration of admission

13 1 OR 2 OR 3 OR 4

14 5 OR 6 OR 7 OR 8 OR 9 OR 10 OR 11 OR 12

15 13 AND 14

**EMBASE:**

1 exp forensic psychiatry/
2 forensic psychiatr*.mp.
3 forensic mental health.mp.
4 ((forensic or secure) adj5 (unit or ward or hospital or department or service or team)).mp.
5 exp "length of stay"/
6 (Length of stay or stay length or LENGTH OF STAY or Duration of stay or Length of admission or Admission Length or Duration of admission or Admission duration).mp.
7 mentally disordered offender*.mp.
8 1 or 2 or 3 or 4 or 7
9 5 or 6
10 8 and 9

**PsycINFO:**

1 exp Forensic Psychiatry/
2 forensic psychiatr*.mp. [mp=title, abstract, heading word, table of contents, key concepts, original title, tests & measures, mesh word]
3 exp Mentally Ill Offenders/
4 forensic mental health.mp.
5 ((forensic or secure) adj5 (unit or ward or hospital or department or service or team)).mp. [mp=title, abstract, heading word, table of contents, key concepts, original title, tests & measures, mesh word]
6 mentally disordered offender*.mp. [mp=title, abstract, heading word, table of contents, key concepts, original title, tests & measures, mesh word]
7 exp Treatment Duration/
8 (Length of stay or stay length or LENGTH OF STAY or Duration of stay or Length of admission or Admission Length or Duration of admission or Admission duration).mp. [mp=title, abstract, heading word, table of contents, key concepts, original title, tests & measures, mesh word]
9 1 or 2 or 3 or 4 or 5 or 6
10 7 or 8
11 9 and 10
