# Supplementary material for: Factors influencing the length of stay in forensic psychiatric settings: a systematic review
Source: BMC Health Serv Res. 2024 Mar 29;24:400. doi: 10.1186/s12913-024-10863-x (PMC10981349; doi:10.1186/s12913-024-10863-x)
Supplement: Supplementary file 2 — Supplementary Material 2 [file 12913_2024_10863_MOESM2_ESM.docx]

## **Supplementary Material 2: Adapted NOS for cross-sectional studies**

**Newcastle-Ottawa Assessment Scale**

(adapted for cross sectional studies)

**Selection: (Maximum 3 stars)**

**1) Representativeness of the sample:**

a) Truly representative of the average in the target population. * (all subjects or random sampling)

b) Somewhat representative of the average in the target population. * (non-random sampling)

c) Selected group of users.

d) No description of the sampling strategy.

**2) Sample size:**

a) Justified and satisfactory.  *

b) Not justified.

**3) Ascertainment of the exposure (risk factor):**

a) Secure record (e.g., surgical record). *

b) Structured interview. (one star) *

c) Written self-report.

d) No description.

e) Other.

**Comparability: (Maximum 2 stars)**

**1) The subjects in different outcome groups are comparable, based on the study design or analysis. Confounding factors are controlled.**

a) The study controls for the most important factor (select one). *

b) The study controls for any additional factor. *

c) The study does not control for any factor.

**Outcome: (Maximum 3 stars)**

**1) Assessment of the outcome:**

a) Independent blind assessment. **

b) Record linkage. **

c) Self report. *

d) No description.

e) Other.

**2) Statistical test:**

a) The statistical test used to analyze the data is clearly described and appropriate, and the measurement of the association is presented, including confidence intervals and the probability level (p value). *

b) The statistical test is not appropriate, not described or incomplete.

**Thresholds for converting the Newcastle-Ottawa scales to AHRQ standards (good, fair, and**

**poor):**

**Good quality**: 3 or 4 stars in selection domain AND 1 or 2 stars in comparability domain AND 2 or 3 stars in outcome/exposure domain

**Fair quality**: 2 stars in selection domain AND 1 or 2 stars in comparability domain AND 2 or 3

stars in outcome/exposure domain

**Poor quality**: 0 or 1 star in selection domain OR 0 stars in comparability domain OR 0 or 1 stars in outcome/exposure domain
