# Supplementary material for: Factors influencing the length of stay in forensic psychiatric settings: a systematic review
Source: BMC Health Serv Res. 2024 Mar 29;24:400. doi: 10.1186/s12913-024-10863-x (PMC10981349; doi:10.1186/s12913-024-10863-x)
Supplement: Supplementary file 3 — Supplementary Material 3 [file 12913_2024_10863_MOESM3_ESM.docx]

## **Supplementary Material 3: Data Extraction Tool - Example**

Date of extraction: 09/09/2022

**Article**: Demographic, Criminogenic, and Psychiatric Factors that Predict Competency Restoration

**Journal**: Journal of the American Academic of Psychiatry and the Law

**Ι. Details of the study**

|  | Description | Location in text |
| --- | --- | --- |
| First Author | Lori H. Colwell | Page 297, under title |
| Year | 2011 | Page 297, under abstract |
| Publication type | Journal article | >> |
| Study period | Not specified |  |
| Aim of the study | Investigate what factors predict competency restoration. In particular what role factors such as treatment refusal, involuntary medication and behavioural management problems played in determining the outcome of restoration effrts. | Page 299, first paragraph (R side) |

1. **Methods**

|  | Description | Location in text |
| --- | --- | --- |
| Study design | Cross-sectional | Page 299, under “Procedure” |
| Population | Forensic inpatients discharged from a maximum-security forensic hospital | Page 299, under “Participants” |
| Setting (incl. Level of security) | Maximum security forensic hospital | Page 299, under “Participants” |
| Country | United States | Page 299, under “Procedure” - mention approval from a Connecticut department |
| Inclusion criteria | Discharge from a forensic maximum-security hospital and having a court-order for restoration of competency to stand trial | Page 299, under “Participants” |
| Exclusion criteria | Nil mentioned |  |
| Recruitment | Nil mentioned |  |
| Sample size | 71 | Page 299, under “Participants” |
| Follow-up period | Nil mentioned |  |
| Statistical analysis | Logistic regression and multiple regression, Pearson correlations | Scattered across the text, includes: Table 6, Table 8 and Page 302, under “Length of Hospitalisation” |

1. **Results - continuous**

| Outcome | Mean | SD | Sample size | Location in text |
| --- | --- | --- | --- | --- |
| Length of stay (days) | 116.3 | 77.0 | 71 | Page 302, under “Length of Hospitalisation” |
| Age | 37.9 | 11.0 | 71 | Page 300, under “Sample Characteristics” |
| Age at first arrest | 23.3 | 9.7 | 71 | Page 301, Table 2 |
| Prior convictions | 4.9 | 7.0 | 71 | >> |
| Prior incarcerations | 2.6 | 3.7 | 71 | >> |
| Maximum exposure | 38.9 | 55.9 | 71 | >> |
| GAF at discharge | 44.3 | 8.4 | 71 | Page 301, Table 3 |
| **Group 1 - Competent** | | | | |
| Prior hospitalisations | 3.06 | 3.67 | 49 | Page 302, Table 4 |
| Prior incarcerations | 2.16 | 2.50 | 49 | >> |
| Prior incompetencies | 1.35 | 0.66 | 49 | >> |
| IQ score | 87.96 | 12.87 | 49 | >> |
| Medications prescribed | 1.91 | 1.01 | 49 | >> |
| Length of stay (days) | 98.92 | 54.54 | 49 | >> |
| GAF at discharge | 47.39 | 6.48 | 49 | >> |
| **Group 2 - Nonrestorable** | | | | |
| Prior hospitalisations | 7.12 | 12.21 | 17 | Page 302, Table 4 |
| Prior incarcerations | 4.29 | 6.09 | 17 | >> |
| Prior incompetencies | 1.94 | 0.90 | 17 | >> |
| IQ score | 69.67 | 13.12 | 17 | >> |
| Medications prescribed | 3.07 | 0.88 | 17 | >> |
| Length of stay (days) | 173.18 | 106.79 | 17 | >> |
| GAF at discharge | 35.65 | 8.08 | 17 | >> |

1. **Results - dichotomous**

| Outcome | No. of events | Sample size | Location in text |
| --- | --- | --- | --- |
| Ethnicity – Caucasian | 28 | 71 | Page 300, Table 1 |
| Ethnicity – African American | 34 | 71 | >> |
| Ethnicity – Hispanic/Latino | 3 | 71 | >> |
| Ethnicity – Arab/Middle Eastern | 1 | 71 | >> |
| Ethnicity – other | 3 | 71 | >> |
| Education level – less than high school | 26 | 71 | >> |
| Education level – high school diploma or equivalent | 22 | 71 | >> |
| Education level – some college | 18 | 71 | >> |
| Education level – college degree | 3 | 71 | >> |
| Education level – post-college education | 2 | 71 | >> |
| Prior involvement with the legal system – yes | 66 | 71 | Page 301, Table 2 |
| Prior conviction for violence – yes | 35 | 71 | >> |
| Controlling offence – felony | 54 | 71 | >> |
| Controlling offence – misdemeanor | 13 | 71 | >> |
| Prior mental health treatment – yes | 62 | 71 | >> |
| Prior episodes of incompetency – none | 45 | 71 | >> |
| Prior episodes of incompetency – one | 14 | 71 | >> |
| Prior episodes of incompetency – two or more | 11 | 71 | >> |
| **Primary** Diagnosis Axis I – psychosis | 36 | 71 | Page 301, Table 3 |
| Diagnosis Axis I – mood/anxiety | 11 | 71 | >> |
| Diagnosis Axis I – substance abuse | 9 | 71 | >> |
| Diagnosis Axis I – cognitive/other | 4 | 71 | >> |
| Diagnosis Axis I – no diagnosis | 7 | 71 | >> |
| **Primary** axis II diagnosis – personality disorder | 21 | 71 | >> |
| Axis II diagnosis – borderline intellectual functioning | 8 | 71 | >> |
| Axis II diagnosis – mental retardation | 5 | 71 | >> |
| Axis II diagnosis – deferred | 16 | 71 | >> |
| Axis II diagnosis – no diagnosis | 21 | 71 | >> |
| Prescribed medications – yes | 53 | 71 | >> |
| Acceptance of medication – accepted voluntarily | 32 | 71 | >> |
| Acceptance of medication – initially resistant but ultimately accepted | 13 | 71 | >> |
| Acceptance of medication – administered involuntarily or not at all | 8 | 71 | >> |
| Seclusion/restraint – none | 61 | 71 | >> |
| Seclusion/restraint – one | 7 | 71 | >> |
| Seclusion/restraint two | 2 | 71 | >> |
| Seclusion/restraint three | 1 | 71 | >> |
| **Group 1 - Competent** | | | |
| Psychosis – yes | 21 | 49 | Page 302, Table 5 |
| Borderline intellectual functioning – yes | 2 | 49 | >> |
| Mental deficiency – yes | 1 | 49 | >> |
| Personality disorder – yes | 18 | 49 | >> |
| Prior incompetencies – none | 37 | 49 | >> |
| Prior incompetencies – one | 7 | 49 | >> |
| Prior incompetencies – more than one | 5 | 49 | >> |
| **Group 2- Nonrestorable** | | | |
| Psychosis – yes | 16 | 17 | Page 302, Table 5 |
| Borderline intellectual functioning – yes | 7 | 17 | >> |
| Mental deficiency – yes | 4 | 17 | >> |
| Personality disorder – yes | 1 | 17 | >> |
| Prior incompetencies – none | 7 | 17 | >> |
| Prior incompetencies – one | 4 | 17 | >> |
| Prior incompetencies – more than one | 6 | 17 | >> |
