# Supplementary material for: Factors influencing the length of stay in forensic psychiatric settings: a systematic review
Source: BMC Health Serv Res. 2024 Mar 29;24:400. doi: 10.1186/s12913-024-10863-x (PMC10981349; doi:10.1186/s12913-024-10863-x)
Supplement: Supplementary file 4 — Supplementary Material 4 [file 12913_2024_10863_MOESM4_ESM.docx]

## **Supplementary Material 4: Variables examined for correlations with length of stay, and the list of studies that found positive, negative, and non-significant correlations**

| **Variables** | **Studies that looked into variables** | **Positive correlation with LOS** | **Negative correlation with LOS** | **NS** |
| --- | --- | --- | --- | --- |
| **Demographics** |  |  |  |  |
| Age | Alexander 2011, Green 1998, Belfrage 2002, Wint 1994, Edwards 2002, Davoren 2015, Gosek 2020, Duke 2018, Ross 2012, Andreasson 2014, Shah 2011, Moran 1999, Pav 2022, Smith 2004, Colwell 2011, Messina 2011 | Wint 1994, Messina 2011 | - | Alexander 2011, Green 1998, Belfrage 2002, Edwards 2002, Davoren 2015, Gosek 2020, Duke 2018, Ross 2012, Andreasson 2014, Shah 2011, Moran 1999, Pav 2022, Smith 2004, Colwell 2011 |
| Female sex | Alexander 2011, Edwards 2002, Davoren 2015, Gosek 2020, Duke 2018, Andreasson 2014, Shah 2011, Pav 2022 | - | Davoren 2015, Pav 2022 | Alexander 2011, Edwards 2002, Gosek 2020, Duke 2018, Andreasson 2014, Shah 2011, Moran 1999 |
| Male sex | Alexander 2011, Edwards 2002, Davoren 2015, Gosek 2020, Duke 2018, Ross 2012, Shah 2011, Moran 1999, Pav 2022 | Davoren 2015, Pav 2022 | - | Alexander 2011, Edwards 2002, Gosek 2020, Duke 2018, Ross 2012, Shah 2011, Moran 1999 |
| White ethnicity | Edwards 2002, Duke 2018, Shah 2011, Moran 1999 | Edwards 2002 | - | Edwards 2002, Duke 2018, Shah 2011, Moran 1999 |
| Afro-Caribbean/African American ethnicity | Edwards 2002, Duke 2018, Shah 2011, Moran 1999 | - | Shah 2011 | Edwards 2002, Duke 2018, Moran 1999 |
| Asian ethnicity | Edwards 2002, Duke 2018, Shah 2011 | - | - | Edwards 2002, Duke 2018, Shah 2011 |
| Mixed ethnic background | Duke 2018, Shah 2011 | - | - | Duke 2018, Shah 2011 |
| Immigrant | Ross 2012, Andreasson 2014 | - | Ross 2012 | Andreasson 2014 |
| **Education/Employment** |  |  |  |  |
| No or special primary education | Eckert 2017, Gosek 2020 | - | - | Eckert 2017, Gosek 2020 |
| Any level of education | Eckert 2017, Gosek 2020, Ross 2012, Pav 2022 | Pav 2022^[[1]](#footnote-1)^ | Ross 2012 | Eckert 2017, Gosek 2020, Pav 2022^a^ |
| No employment history | Eckert 2017, Gosek 2020, Moran 1999, Pav 2022 | Pav 2022 | - | Eckert 2017, Gosek 2020, Moran 1999 |
| Any employment history | Eckert 2017, Gosek 2020, Ross 2012, Andreasson 2014, Moran 1999, Pav 2022 | - | Ross 2012, Moran 1999, Pav 2022 | Eckert 2017, Gosek 2020, Ross 2012, Moran 1999, Pav 2022 |
| **Family Status** |  |  |  |  |
| Married/engaged | Ross 2012, Andreasson 2014, Moran 1999, Pav 2022 | - | Ross 2012, Pav 2022 | Andreasson 2014, Moran 1999 |
| Single or in unstable relationship | Ross 2012, Shah 2011, Moran 1999, Pav 2022 | Pav 2022 | - | Ross 2012, Shah 2011, Moran 1999 |
| Widowed | Ross 2012, Moran 1999 | - | - | Ross 2012, Moran 1999 |
| Divorced | Ross 2012, Moran 1999 | - | Ross 2012 | Moran 1999 |
| Parent | Ross 2012, Andreasson 2014 | - | Andreasson 2014 | Ross 2012 |
| **Admission Source** |  | - | - |  |
| Community | Edwards 2002, Duke 2018 | Duke 2018 | - | Edwards 2002 |
| High-security hospital | Edwards 2002, Duke 2018 | Duke 2018 | - | Edwards 2002 |
| Medium-secure unit | Duke 2018, Pav 2022 | Pav 2022 | - | Duke 2018 |
| Prison or court | Edwards 2002, Duke 2018 | Duke 2018 | - | Edwards 2002 |
| **Diagnosis** |  |  |  |  |
| Adjustment disorder | Davoren 2015, Rodenhauser 1988 | - | Davoren 2015 | Rodenhauser 1988 |
| Affective disorders | Green 1998, Gosek 2020, Andreasson 2014, Shah 2011, Pav 2022, Rodenhauser 1988 | Andreasson 2014 | Shah 2011 | Green 1998, Gosek 2020, Pav 2022, Rodenhauser 1988 |
| ASD^[[2]](#footnote-2)^ / Pervasive developmental disorders | Alexander 2011, Verstegen 2017, Eckert 2017, Pav 2022 | - | - | Alexander 2011, Verstegen 2017, Eckert 2017, Pav 2022 |
| Anxiety disorders | Andreasson 2014, Shah 2011, Pav 2022, Rodenhauser 1988 | - | - | Andreasson 2014, Shah 2011, Pav 2022, Rodenhauser 1988 |
| Bipolar | Alexander 2011, Moran 1999, Smith 2004 | - | Alexander 2011 | Moran 1999, Smith 2004 |
| Depression | Alexander 2011, Moran 1999 | - | - | Moran 1999, Alexander 2011 |
| Impulse control disorder | Andreasson 2014, Rodenhauser 1988 | - | - | Andreasson 2014, Rodenhauser 1988 |
| Intellectual or Learning Disability | Gosek 2020, Shah 2011, Pav 2022, Chester 2018 | Pav 2022 | Chester 2018 | Gosek 2020, Shah 2011 |
| Organic mental disorders | Gosek 2020, Shah 2011, Pav 2022, Rodenhauser 1988 | - | - | Gosek 2020, Shah 2011, Pav 2022, Rodenhauser 1988 |
| Personality disorders | Alexander 2011, Belfrage 2002, Edwards 2002, Long 2012, Gosek 2020, Andreasson 2014, Shah 2011, Pav 2022, Rodenhauser 1988 | - | Alexander 2011, Long 2012, Rodenhauser 1988 | Belfrage 2002, Edwards 2002, Gosek 2020, Andreasson 2014, Shah 2011, Pav 2022 |
| Personality disorders - Cluster B | Ross 2012, Shah 2011 | Ross 2012 | - | Shah 2011 |
| Sexual preference disorder | Eckert 2017, Ross 2012, Pav 2022, Rodenhauser 1988 | Pav 2022 | - | Ross 2012, Eckert 2017, Rodenhauser 1988 |
| Scizophrenia-broad | Eckert 2017, Davoren 2015, Long 2012, Moran 1999, Rodenhauser 1988, Messina 2011 | Eckert 2017, Long 2012, Rodenhauser 1988, Messina 2011 | - | Davoren 2015, Moran 1999 |
| Schizophrenia | Edwards 2002, Gosek 2020, Smith 2004 | Gosek 2020 | - | Edwards 2002, Smith 2004 |
| Schizoaffective Disorder | Gosek 2020, Smith 2004 | Gosek 2020 | - | Smith 2004 |
| Substance use disorders | Alexander 2011, Green 1998, Eckert 2017, Ross 2012, Shah 2011, Moran 1999, Pav 2022, Rodenhauser 1988 | - | Eckert 2017^[[3]](#footnote-3)^, Pav 2022^[[4]](#footnote-4)^ | Alexander 2011, Green 1998, Eckert 2017^b^, Ross 2012, Shah 2011, Moran 1999, Rodenhauser 1988 |
| Substance-induced Psychosis | Gosek 2020, Smith 2004 | - | - | Gosek 2020, Smith 2004 |
| Psychosis-related | Alexander 2011, Green 1998, Belfrage 2002, Gosek 2020, Ross 2012, Andreasson 2014, Shah 2011, Moran 1999, Pav 2022, Smith 2004, Rodenhauser 1988 | Green 1998, Andreasson 2014, Shah 2011, Pav 2022 | Ross 2012 | Alexander 2011, Belfrage 2002, Gosek 2020, Moran 1999, Smith 2004, Rodenhauser 1988 |
| **Scores** |  |  |  |  |
| IQ^[[5]](#footnote-5)^ score | Wint 1994, Eckert 2017, Colwell 2011 | - | Colwell 2011 | Wint 1994, Eckert 2017 |
| GAF^[[6]](#footnote-6)^ Score | Andreasson 2014, Colwell 2011, Messina 2011 | - | Andreasson 2014, Colwell 2011, Messina 2011 |  |
| DUNDRUM-1^[[7]](#footnote-7)^ Total Score | Eckert 2017, Davoren 2015 | Eckert 2017, Davoren 2015 | - |  |
| HCR20^[[8]](#footnote-8)^ Score - Clinical | Belfrage 2002, Eckert 2017, Davoren 2015 | Eckert 2017 | - | Belfrage 2002, Davoren 2015 |
| HCR20 Score - Historical | Belfrage 2002, Eckert 2017, Davoren 2015 | - | - | Belfrage 2002, Eckert 2017, Davoren 2015 |
| HCR20 Score - Risk | Belfrage 2002, Eckert 2017, Davoren 2015 | Belfrage 2002, Eckert 2017 | - | Davoren 2015 |
| HCR20 Score - Total | Belfrage 2002, Eckert 2017, Davoren 2015, Smith 2004 | Belfrage 2002 | - | Eckert 2017, Davoren 2015, Smith 2004 |
| **Forensic History** |  |  |  |  |
| Age at first conviction | Edwards 2002, Eckert 2017, Andreasson 2014, Shah 2011 | - | - | Edwards 2002, Eckert 2017, Andreasson 2014, Shah 2011 |
| Any previous convictions | Shah 2011, Moran 1999, Rodenhauser 1988 | - | - | Shah 2011, Moran 1999, Rodenhauser 1988 |
| Number of previous convictions/sentences/incarcerations | Andreasson 2014, Smith 2004, Colwell 2011 | - | - | Andreasson 2014, Smith 2004, Colwell 2011 |
| Presence of previous major offence | Alexander 2011, Edwards 2002, Eckert 2017, Shah 2011 | - | - | Alexander 2011, Edwards 2002, Eckert 2017, Shah 2011 |
| History of previous incompetencies | Moran 1999, Colwell 2011 | - | - | Moran 1999, Colwell 2011 |
| Amount of previous forensic treatment | Gosek 2020, Andreasson 2014, Shah 2011 | Andreasson 2014 | - | Gosek 2020, Shah 2011 |
| **Psychiatric History** |  |  |  |  |
| Previous psychiatric treatment | Edwards 2002, Gosek 2020, Andreasson 2014, Shah 2011, Dell 1987, Moran 1999, Pav 2022 | Andreasson 2014, Shah 2011, Dell 1987, Pav 2022 | - | Edwards 2002, Gosek 2020, Moran 1999 |
| Duration of mental illness in years | Gosek 2020, Smith 2004 | Gosek 2020 | - | Smith 2004 |
| Number of previous psychiatric admissions | Shah 2011, Pav 2022, Smith 2004, Rodenhauser 1988, Colwell 2011 | Rodenhauser 1988 | - | Shah 2011, Pav 2022, Smith 2004, Colwell 2011 |
| **Other history** |  |  |  |  |
| Family history of mental illness | Eckert 2017, Gosek 2020, | - | - | Eckert 2017, Gosek 2020, |
| Any past history of abuse | Alexander 2011, Eckert 2017, McKenna 2019 | Eckert 2017^[[9]](#footnote-9)^ | - | Alexander 2011, Eckert 2017^e^ McKenna 2019 |
| **Index Offence** |  |  |  |  |
| Age at index offence | Eckert 2017, Moran 1999 | - | Moran 1999 | Eckert 2017 |
| Index offence of sexual nature | Edwards 2002, Eckert 2017, Gosek 2020, Ross 2012. Dell 1987, Moran 1999, Smith 2004, Messina 2011 | Ross 2012, Dell 1987^[[10]](#footnote-10)^, Messina 2011 | - | Edwards 2002, Eckert 2017, Gosek 2020, Dell 1987^f^, Moran 1999, Smith 2004 |
| Index offence of homicide or attempted homicide | Green 1998, Edwards 2002, Gosek 2020, Ross 2012, Dell 1987, Moran 1999 | Green 1998, Edwards 2002^[[11]](#footnote-11)^, Gosek 2020, Ross 2012, Dell 1987^f^, | - | Edwards 2002^g^, Dell 1987^f^, Moran 1999 |
| Major index offence | Green 1998, Edwards 2002, Eckert 2017, Long 2012, Gosek 2020, Ross 2012, Andreasson 2014, Shah 2011, Dell 1987, Moran 1999, Pav 2022, Smith 2004, Rodenhauser 1988, Colwell 2011, Messina 2011 | Green 1998, Edwards 2002, Long 2012, Gosek 2020, Ross 2012, Andreasson 2014, Dell 1987, Rodenhauser 1988, Messina 2011 | Ross 2012, Shah 2011, Dell 1987 | Edwards 2002, Eckert 2017, Gosek 2020, Dell 1987, Moran 1999, Pav 2022, Smith 2004, Colwell 2011 |
| Minor index offence | Edwards 2002, Long 2012, Ross 2012, Shah 2011, Pav 2022, Smith 2004, Rodenhauser 1988, Colwell 2011 | - | Long 2012, Ross 2012 | Edwards 2002, Shah 2011, Pav 2022, Smith 2004, Rodenhauser 1988, Colwell 2011 |
| Nil offence | Green 1998, Edwards 2002, Smith 2004 | - | - | Green 1998, Edwards 2002, Smith 2004 |
| Continuous index offence | Gosek 2020 | - | - | Gosek 2020 |
| Index offence under influence of alcohol/psychoactive substances | Gosek 2020, Pav 2022 | - | Pav 2022 | Gosek 2020, |
| **Institutional Aggression** |  |  |  |  |
| Violence to others | Verstegen 2017, Davoren 2015, Gosek 2020, Andreasson 2014 | Verstegen 2017 | - | Davoren 2015, Gosek 2020, Andreasson 2014 |
| Number of acts of violence | Verstegen 2017, Andreasson 2014 | - | Verstegen 2017 | Andreasson 2014 |
| Presence of violence to self | Davoren 2015, Andreasson 2014 | - | - | Davoren 2015, Andreasson 2014 |
| Amount of absconding | Eckert 2017, Andreasson 2014 | Eckert 2017 | - | Andreasson 2014 |
| Seclusion and/or restraint | Alexander 2011, Griffiths 2018, Davoren 2015, Rodenhauser 1988, Colwell 2011 | Rodenhauser 1988 | - | Alexander 2011, Griffiths 2018, Davoren 2015, Colwell 2011 |
| **Legal Category/Status** |  |  |  |  |
| Legal category - Mental Illness | Wint 1994, Edwards 2002, Dell 1987 | Wint 1994 | - | Edwards 2002, Dell 1987 |
| Legal category - Psychopathic Disorder | Wint 1994, Edwards 2002, Dell 1987 | - | Wint 1994 | Edwards 2002, Dell 1987 |
| Legal status - civil | Edwards 2002, Davoren 2015, Brown 2009, Duke 2018, Shah 2011, Dell 1987 | - | Duke 2018 | Edwards 2002, Davoren 2015, Brown 2009, Shah 2011, Dell 1987 |
| Legal status - prison transfer | Edwards 2002, Brown 2009, Duke 2018, Shah 2011, Dell 1987 | - | Duke 2018 | Edwards 2002, Brown 2009, Shah 2011, Dell 1987 |
| Legal status - criminal section | Alexander 2011, Edwards 2002, Brown 2009, Shah 2011, Dell 1987 | Shah 2011 | - | Alexander 2011, Edwards 2002, Brown 2009, Dell 1987 |
| Legal status - criminal section with restrictions | Alexander 2011, Green 1998, Edwards 2002, Brown 2009, Duke 2018, Shah 2011, Dell 1987 | Green 1998, Brown 2009, Duke 2018, Shah 2011 | - | Alexander 2011, Edwards 2002, Dell 1987 |
| Legal status - no criminal responsibility | Edwards 2002, Davoren 2015, Ross 2012, Dell 1987 | - | Ross 2012 | Edwards 2002, Davoren 2015, Dell 1987 |
| Legal status - remand order | Edwards 2002, Davoren 2015, Shah 2011 | Davoren 2015 | - | Edwards 2002, Shah 2011 |
| **Treatment** |  |  |  |  |
| Psychotherapy engagement | Long 2012, Moulden 2020 | - | Long 2012 | Moulden 2020 |
| Attendance of groups while admitted | Colwell 2011, Messina 2011 | - | Messina 2011 | Colwell 2011 |

1. Positive correlation of LOS with “any professional education”, NS correlation with “secondary or higher education” [↑](#footnote-ref-1)
2. ASD: Autism Spectrum Disorder [↑](#footnote-ref-2)
3. Negative correlation of LOS with recent substance use, NS correlation with lifetime substance dependence disorder [↑](#footnote-ref-3)
4. Substance use including alcohol [↑](#footnote-ref-4)
5. IQ: Intelligence Quotient [↑](#footnote-ref-5)
6. GAF: Global Assessment of Functioning [↑](#footnote-ref-6)
7. DUNDRUM-1: Dangerousness, Understanding, Recovery and Urgency Manual – Triage Security Items (92) [↑](#footnote-ref-7)
8. HCR20: Historical, Clinical and Risk Management [↑](#footnote-ref-8)
9. Positive correlation between length of stay and history of emotional neglect, non-significant with history of sexual abuse [↑](#footnote-ref-9)
10. Positive in cohort of “psychopaths” and NS in cohort of “non-psychopaths” [↑](#footnote-ref-10)
11. Positive in cohort of 5 years, NS in cohort of 2 years [↑](#footnote-ref-11)
